# Supplementary material for: MIWI N-terminal arginines orchestrate generation of functional pachytene piRNAs and spermiogenesis
Source: Nucleic Acids Res. 2024 Mar 23;52(11):6558–70. doi: 10.1093/nar/gkae193 (PMC11194079; doi:10.1093/nar/gkae193)
Supplement: gkae193_Supplemental_Files [file gkae193_supplemental_files.zip › Revised_Supplement_2-23-2024.pdf]

SUPPLEMENTARY INFORMATION

MIWI N-terminal arginines orchestrate generation of functional pachytene piRNAs and spermiogenesis

Nicholas Vrettos [1, *], Jan Oppelt [1, *], Ansgar Zoch [2, *], Paraskevi Sgourdou [1], Haruka Yoshida [2], Brian Song[1], Ryan Fink[1], Dónal O'Carroll [2, 3 ‡] and Zissimos Mourelatos [1, ‡]

[1] Department of Pathology and Laboratory Medicine, Division of Neuropathology, Perelman School of Medicine, University of Pennsylvania, Philadelphia, Pennsylvania 19104, USA

[2] Centre for Regenerative Medicine, Institute for Stem Cell Research, School of Biological Sciences

[3] Wellcome Centre for Cell Biology, University of Edinburgh, Edinburgh, UK

* These authors contributed equally to this work: NV, JO, AZ

‡ Correspondence to: donal.ocarroll@ed.ac.uk and Zissimos.Mourelatos@pennmedicine.upenn.edu

Supplementary Figures 1 to 10

Supplementary Tables 1 to 15

SUPPLEMENTARY FIGURE LEGENDS

Supplementary Figure 1 Generation and characterization of the *Miwi*^{RK} allele

A. Schematic representation of the mouse *Miwi* (*Piwil1*) locus, along with a small guide RNA (sgRNA) that targets part of exon 3 encoding the N-terminus of MIWI. **B.** Recombination strategy for generating the *Miwi*^{RK} allele. Single-strand (ss) DNA donor oligonucleotide containing the designed mutations flanked by 5´ and 3´homology arms (HA). **C.** Sequencing trace of exon 3 area harboring the RK mutations. **D.** Representative genotyping results for *Miwi*^{WT}, *Miwi*$^{+/RK}$ and *Miwi*$^{RK/RK}$ animals.

Supplementary Figure 2 Characteristics of MILI- and MIWI- bound piRNAs in *Miwi*$^{+/RK}$ and *Miwi*$^{RK/RK}$

A. Base composition of MILI and MIWI piRNAs from *Miwi*$^{+/RK}$ and *Miwi*$^{RK/RK}$ P24 testes mapping to pachytene clusters. **B.** Length distribution off all piRNAs (left) or piRNA derived exclusively from pachytene clusters (right). Lengths between 24 to 32 nucleotides are highlighted with dotted lines. **C.** Histogram of median 3' end length difference for piRNAs with the same starting position (5' end) between *Miwi*$^{+/RK}$ and *Miwi*$^{RK/RK}$ piRNAs bound to MILI (top) or MIWI (bottom). y axis shows number of piRNAs and x axis shows 3' end length differences, binned per nucleotide. A value of zero indicates identical 3' ends; positive numbers indicate piRNAs whose 3' ends are longer in *Miwi*$^{+/RK}$; negative numbers indicate piRNAs whose 3' ends are longer in *Miwi*$^{RK/RK}$.

Supplementary Figure 3 Differential expression of MILI piRNAs derived from piRNA clusters, repeats, intergenic areas and introns between *Miwi*$^{+/RK}$ and *Miwi*$^{RK/RK}$

Differential expression analysis of MILI piRNAs mapping to indicated genomic features (**A**) and differential expression analysis of indicated genomic features (**B**), between *Miwi*$^{+/RK}$ and *Miwi*$^{RK/RK}$ visualized by Volcano (left) and MA (right) plots; red, adjusted p-value < 0.05 and fold-change >=2; blue, adjusted p-value < 0.05 and fold-change <=-2; grey, adjusted p-value >= 0.05 and/or fold-change > -2 < 2.

Supplementary Figure 4 Differential expression of MILI piRNAs derived from exons, 5' Untranslated Regions (5'-UTR), Coding Sequences (CDS) and 3' Untranslated Regions (3'-UTR) between *Miwi*$^{+/RK}$ and *Miwi*$^{RK/RK}$

Differential expression analysis of MILI piRNAs mapping to indicated genomic features (**A**) and differential expression analysis of indicated genomic features (**B**), between *Miwi*$^{+/RK}$ and *Miwi*$^{RK/RK}$ visualized by Volcano (left) and MA (right) plots; red, adjusted p-value < 0.05 and fold-change >=2; blue, adjusted p-value < 0.05 and fold-change <=-2; grey, adjusted p-value >= 0.05 and/or fold-change > -2 < 2.

Supplementary Figure 5 Differential expression of MIWI piRNAs derived from piRNA clusters, repeats, intergenic areas and introns between *Miwi*$^{+/RK}$ and *Miwi*$^{RK/RK}$

Differential expression analysis of MIWI piRNAs mapping to indicated genomic features (**A**) and differential expression analysis of indicated genomic features (**B**), between *Miwi*$^{+/RK}$ and *Miwi*$^{RK/RK}$ visualized by Volcano (left) and MA (right) plots; red, adjusted p-value < 0.05 and fold-change >=2; blue, adjusted p-value < 0.05 and fold-change <=-2; grey, adjusted p-value >= 0.05 and/or fold-change > -2 < 2.

Supplementary Figure 6 Differential expression of MIWI piRNAs derived from exons, 5' Untranslated Regions (5'-UTR), Coding Sequences (CDS) and 3' Untranslated Regions (3'-UTR) between *Miwi*$^{+/RK}$ and *Miwi*$^{RK/RK}$

Differential expression analysis of MIWI piRNAs mapping to indicated genomic features (**A**) and differential expression analysis of indicated genomic features (**B**), between *Miwi*$^{+/RK}$ and *Miwi*$^{RK/RK}$ visualized by Volcano (left) and MA (right) plots; red, adjusted p-value < 0.05 and fold-change >=2; blue, adjusted p-value < 0.05 and fold-change <=-2; grey, adjusted p-value >= 0.05 and/or fold-change > -2 < 2.

Supplementary Figure 7 Differential expression of MILI and MIWI piRNAs, grouped by the same 5′ end, between *Miwi*$^{+/RK}$ and *Miwi*$^{RK/RK}$

Differential expression analysis of MILI piRNAs (**A**) and MIWI piRNAs (**B**), grouped by the same 5′ end, between *Miwi*$^{+/RK}$ and *Miwi*$^{RK/RK}$ visualized by Volcano (top) and MA (bottom) plots; red, adjusted

p-value < 0.05 and fold-change >=2; blue, adjusted p-value < 0.05 and fold-change <=-2; grey, adjusted p-value >= 0.05 and/or fold-change > -2 < 2.

Supplementary Figure 8 Ping-pong of MILI and MIWI piRNAs in exons, in *Miwi*$^{+/RK}$ and *Miwi*$^{RK/RK}$

5'-5' distance (ping-pong) analyses and Z-scores of MILI-bound (**A**) and MIWI-bound (**B**) piRNAs mapping to exons.

Supplementary Figure 9 Heterotypic, MILI-MIWI ping-pong in *Miwi*$^{+/RK}$ and *Miwi*$^{RK/RK}$

5'-5' distance (ping-pong) analyses and Z-scores between MILI and MIWI piRNAs mapping to pachytene clusters.

Supplementary Figure 10 Acrosome malformation in *Miwi*$^{RK/RK}$

Electron micrographs of *Miwi*$^{+/RK}$ and *Miwi*$^{RK/RK}$ round spermatids. Nu, nucleus; Ap, acroplaxosome; Av, acrosomal granule; Ag, acrosomal vesicle; Go, Golgi apparatus. Scale bars, 1 µm.

Supplementary Figure 1

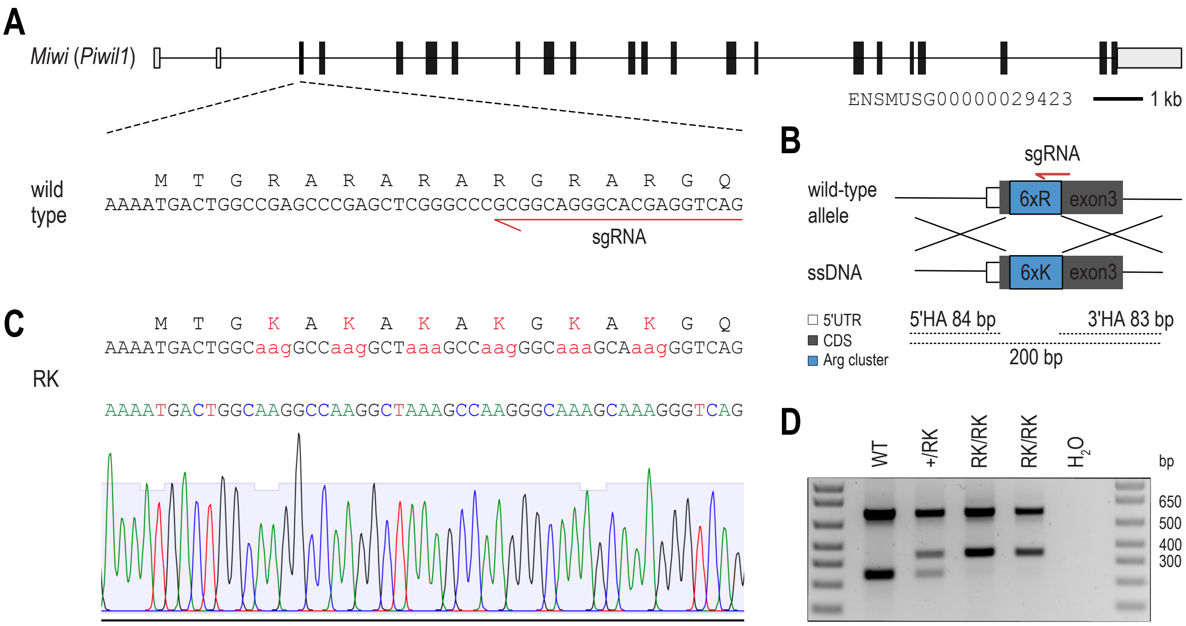

Supplementary Figure 2

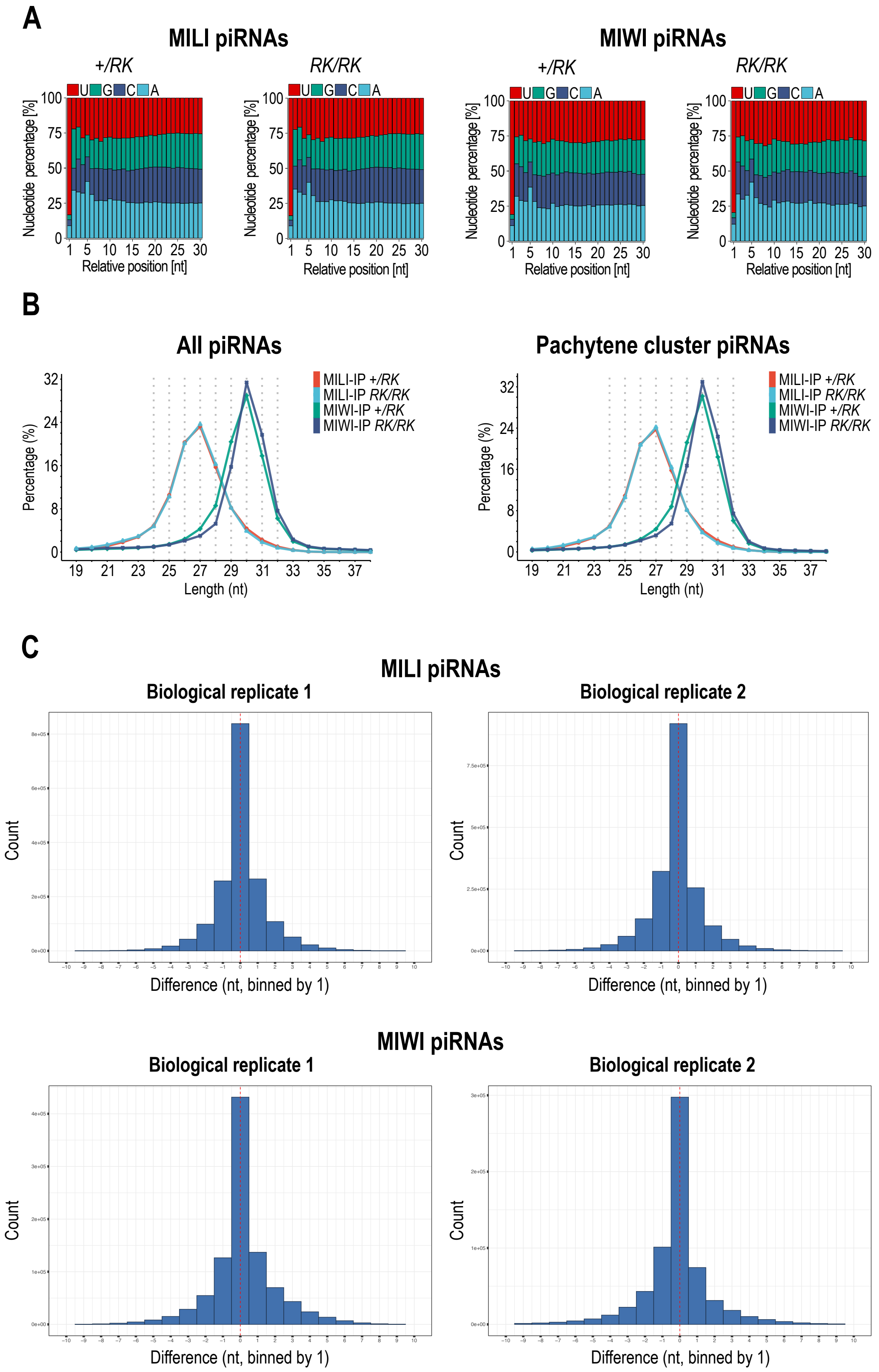

Supplementary Figure 3

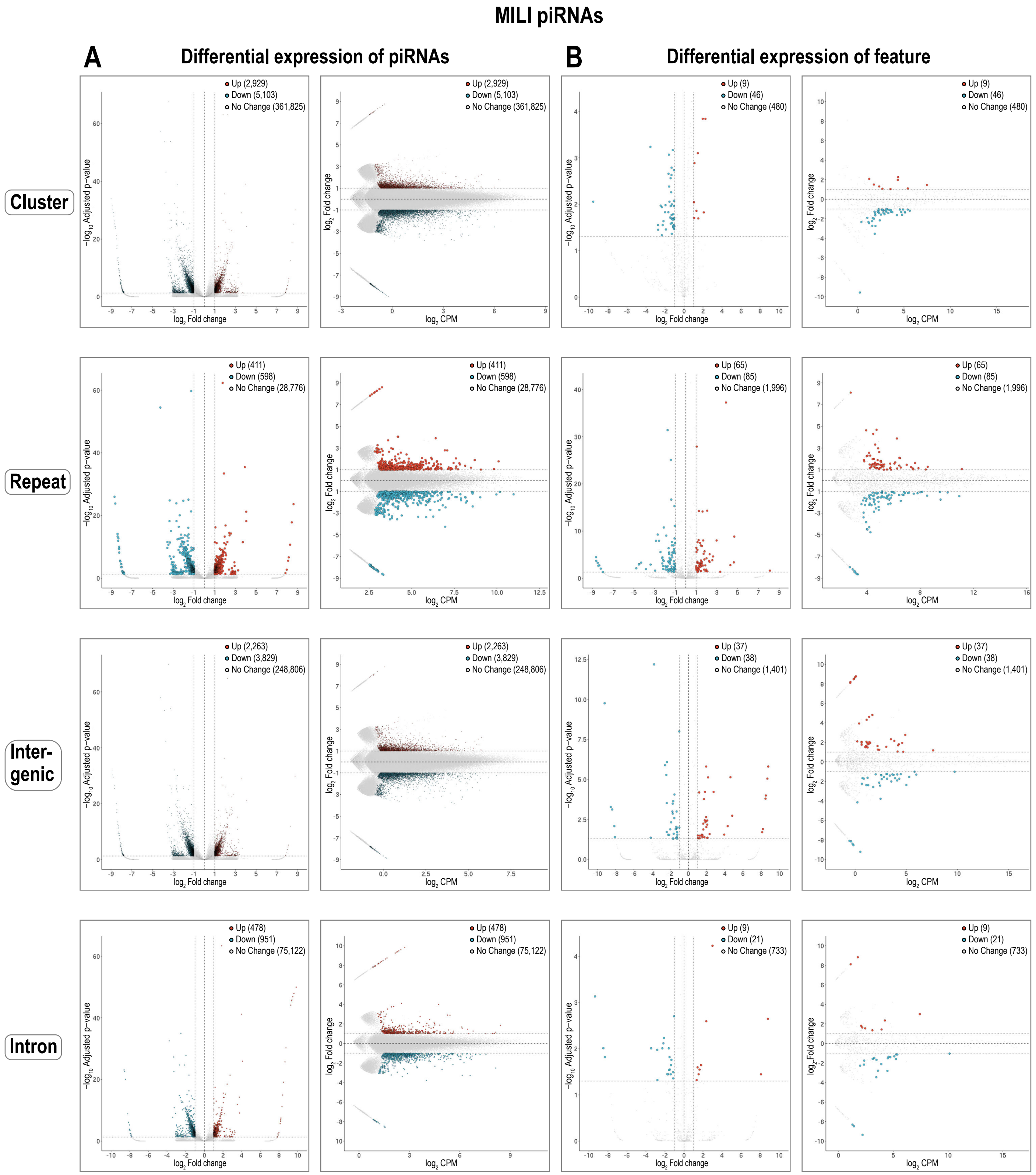

Supplementary Figure 4

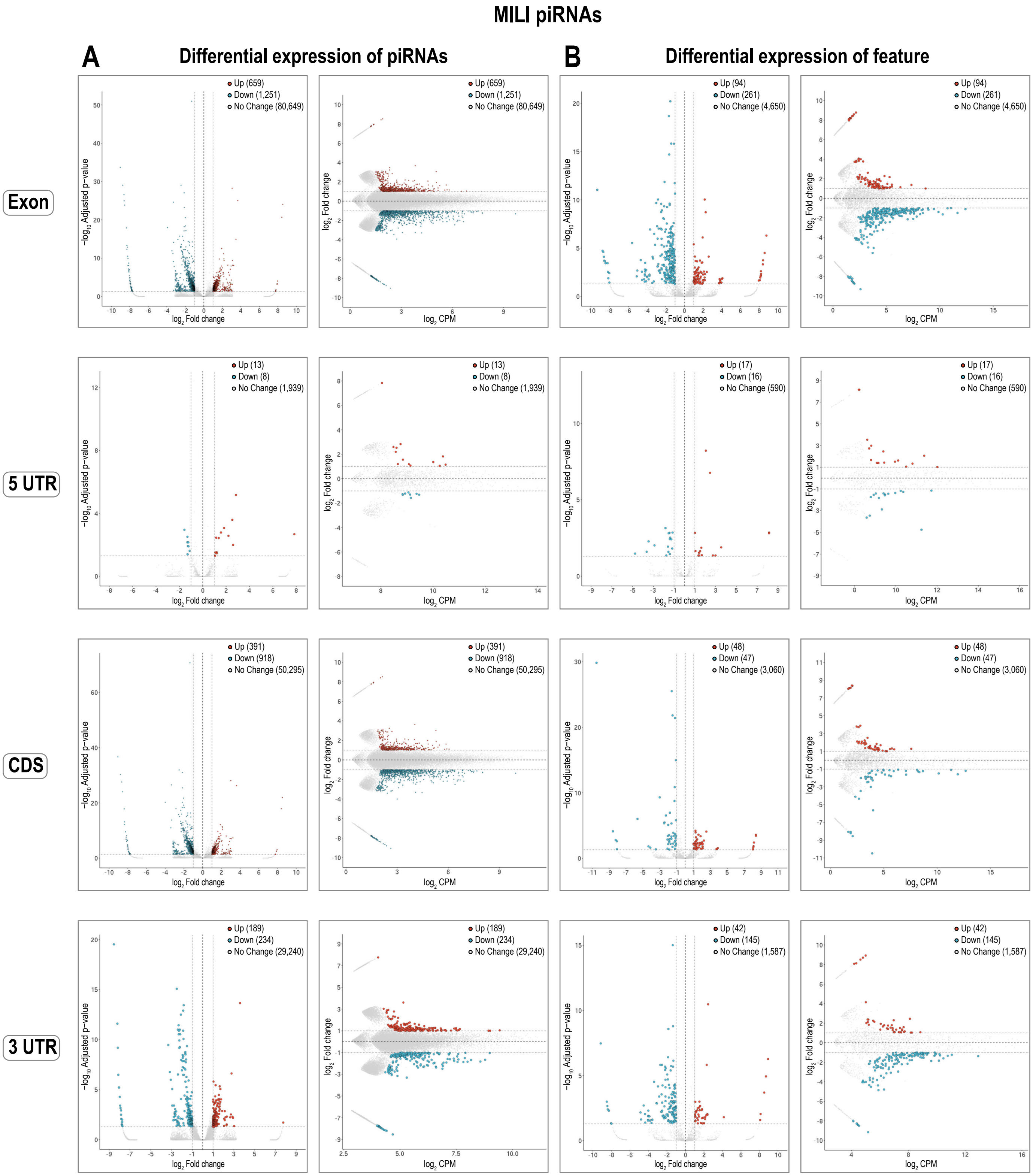

Supplementary Figure 5

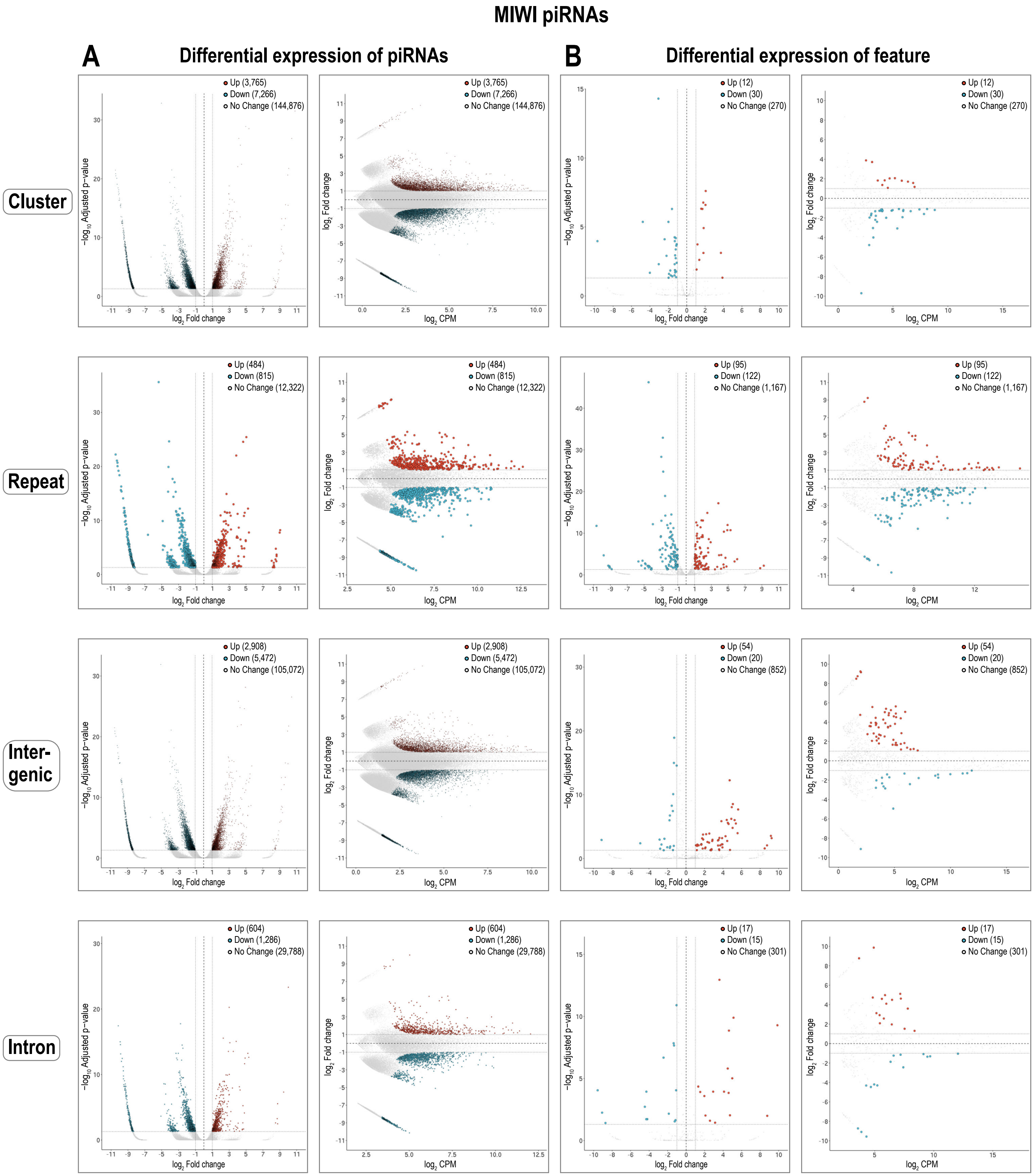

Supplementary Figure 6

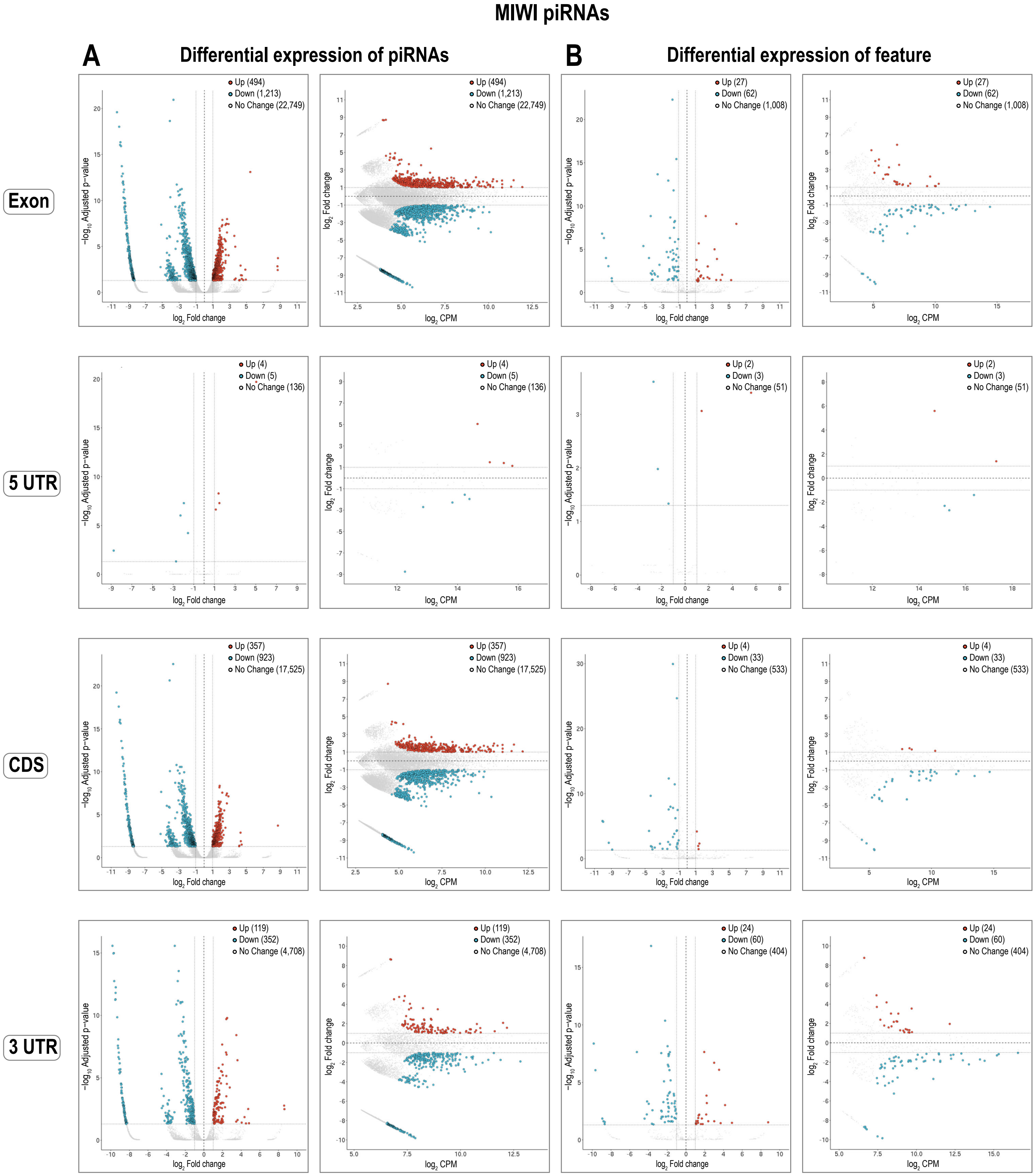

Supplementary Figure 7

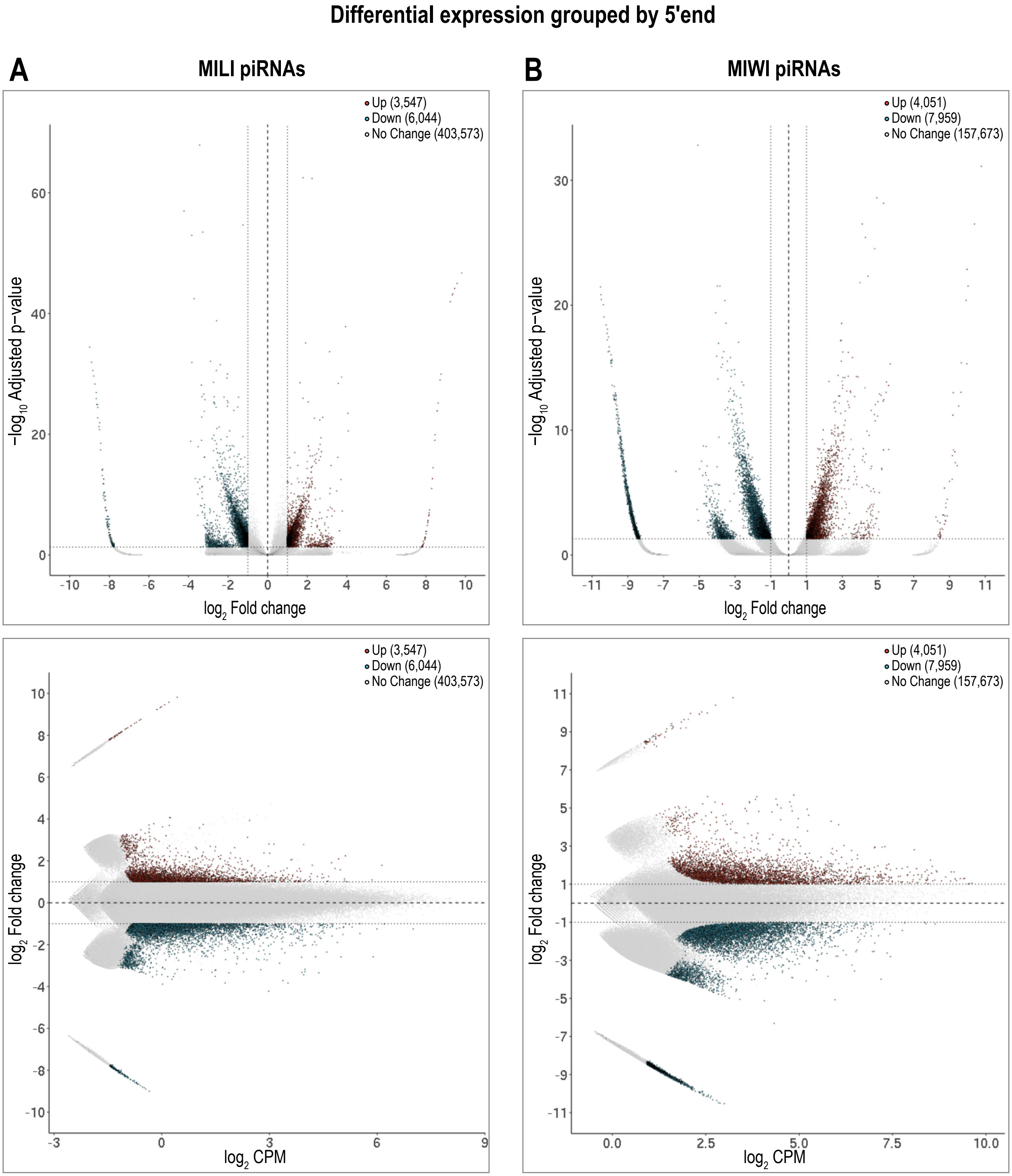

Supplementary Figure 8

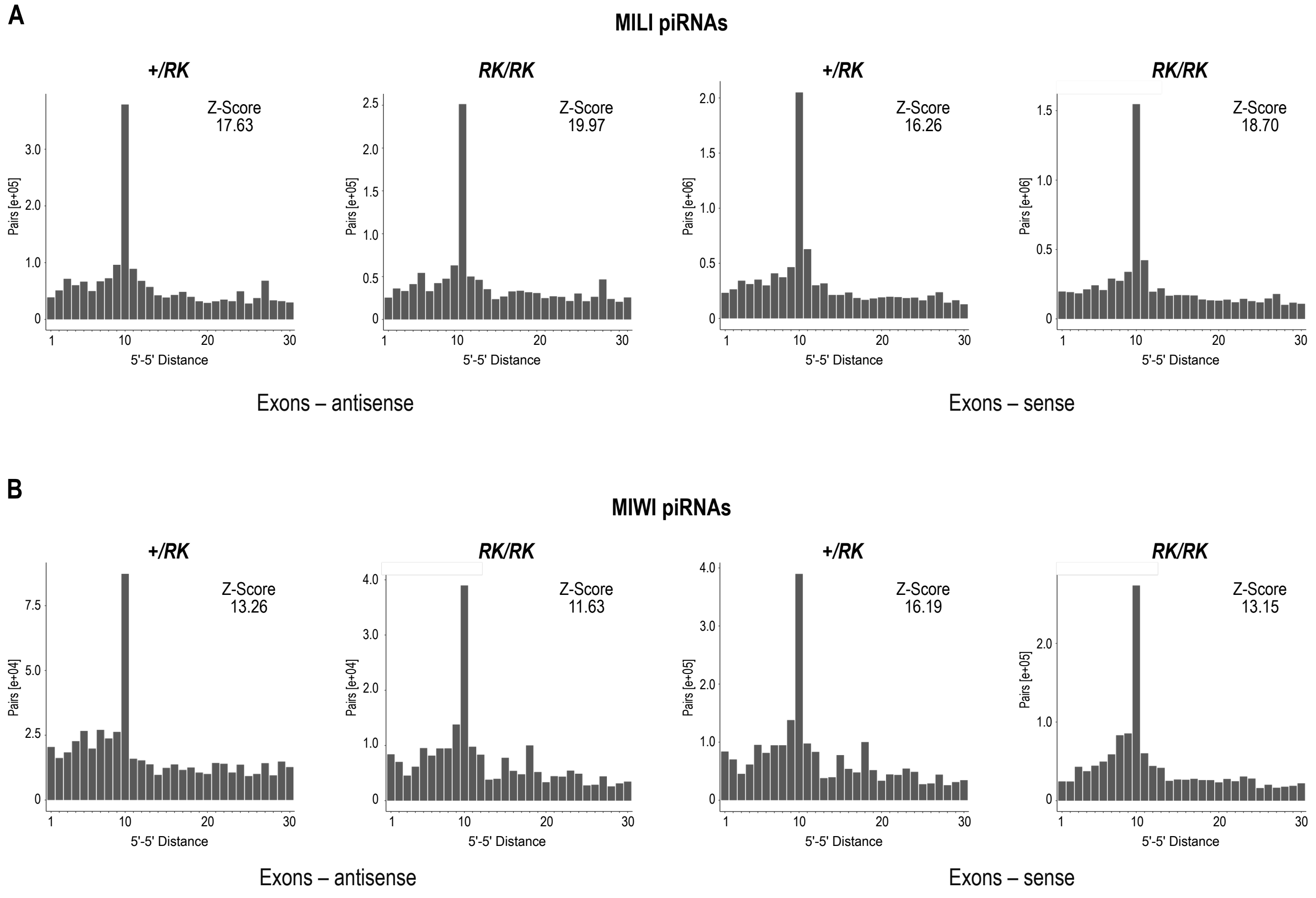

Supplementary Figure 9

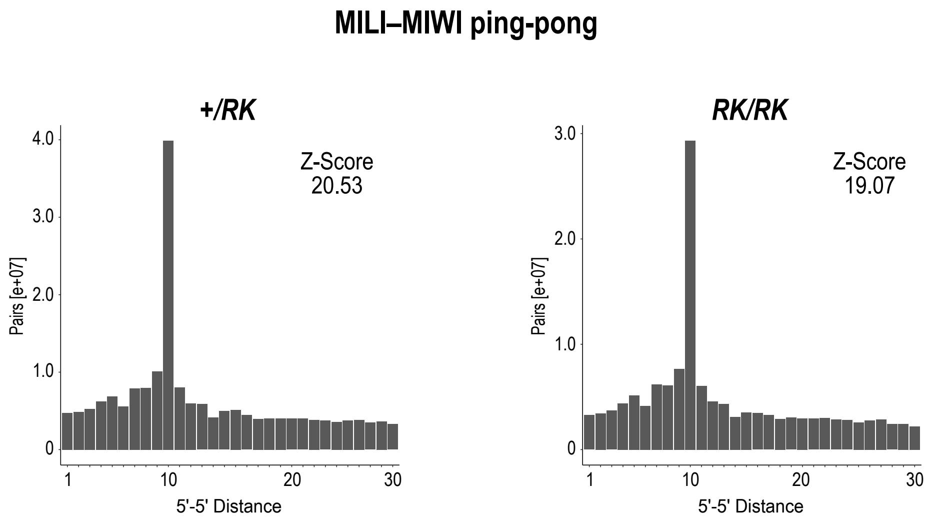

Supplementary Figure 10

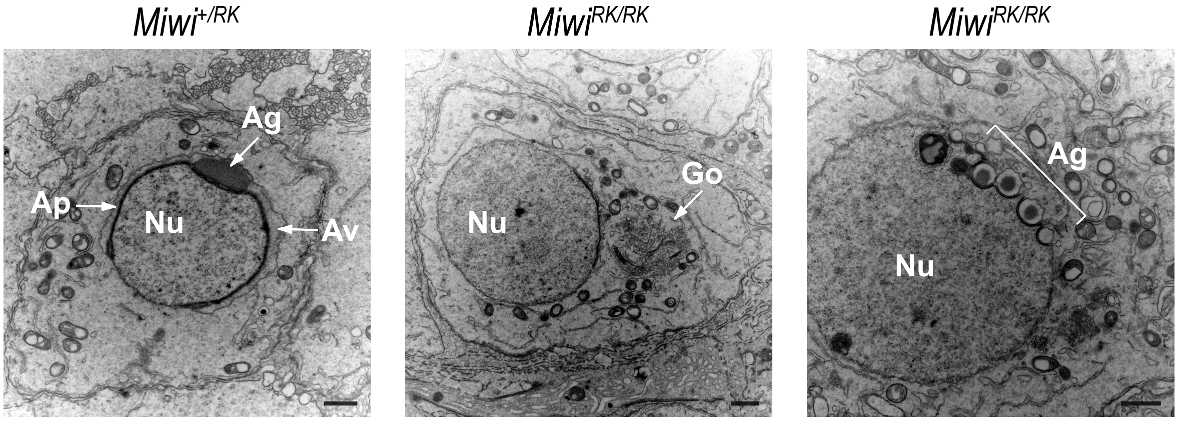

SUPPLEMENTARY TABLE LEGENDS (Excel files provided separately)

Supplementary Table 1: $Miwi^{RK/RK}$ male mice present with a fully penetrant infertility phenotype

Supplementary Table 2: Testes of $Miwi^{RK/RK}$ adult mice are significantly smaller than those of wild-type littermates

Supplementary Table 3: Fraction of piRNAs in ping-pong pairs mapping to gene exons in antisense orientation, excluding repeat mapped

Supplementary Table 4: Fraction of piRNAs in ping-pong pairs mapping to gene exons in sense orientation, excluding repeat mapped

Supplementary Table 5: Fraction of piRNAs in ping-pong pairs mapping to pachytene clusters, excluding repeat mapped

Supplementary Table 6: Fraction of piRNAs in ping-pong pairs mapping to repeats inside gene exons

Supplementary Table 7: Fraction of piRNAs in ping-pong pairs mapping to repeats inside pachytene clusters

Supplementary Table 8: Fraction of piRNAs in ping-pong pairs mapping to repeats outside pachytene clusters

Supplementary Table 9: Transposon differential expression

Supplementary Table 10: Transcript differential expression

Supplementary Table 11: Differential ribosome occupancy

Supplementary Table 12: Comparison of differential expression between $Miwi^{RK/RK}$ vs. $Miwi^{+/RK}$ and $Tdrd6^{-/-}$ vs. $Tdrd6^{+/-}$

Supplementary Table 13: List of antibodies used for immunofluorescence, immunoprecipitations and western blots

Supplementary Table 14: List of oligonucleotides used for piRNA library construction

Supplementary Table 15: List of primers used for RT-qPCR analysis
